# Age-related changes in the vasculature of the dermis of the upper lip vermilion

**DOI:** 10.18632/aging.101996

**Published:** 2019-06-06

**Authors:** Takamasa Gomi, Toru Imamura

**Affiliations:** 1 Cell Regulation Laboratory, Bionics Program, Tokyo University of Technology Graduate School of Bionics, Computer and Media Science, Hachioji, Japan; 2 Frontier Research Center, POLA Chemical Industries Inc., Yokohama, Japan

**Keywords:** lip, vermilion, labium oris, aging, blood vessel, rete ridge

## Abstract

Lip redness is unique to humans and creates an important facial impression, but this redness decreases with age. Here, using histological and immunohistological staining of human upper lip vermilion from donors of different ages, we investigated blood vessels in the upper lip dermis and age-dependent histological changes. We found that both total vessel area in the dermis and vessel number in the upper dermis decreased with aging. Moreover, vessel number in the upper dermis correlated positively with development of rete ridges, which flattened with age, despite no significant change in the thickness of the stratified squamous epithelium. These findings suggest that age-related reductions in lip redness result from a decrease of blood vessels, which in turn leads to a flattening of the epithelium caused by the loss of rete ridges. This is the first study to histologically demonstrate age-related reductions in blood vessels in the lip. Our results provide an opportunity for enhancing blood flow/vascularization to improve the aesthetic appearance of the lips in the elderly.

## Introduction

The highly visible reddish vermilion of the labium oris (lip surrounding the mouth) is unique to humans. Although the area of vermilion makes up only a small fraction of the total facial area, the redness of the vermilion profoundly affects the impressions of others. A redder vermilion lightens the perceived complexion [1], and a red vermilion increases the facial attractiveness of female Caucasian faces [2,3]. The redness of the vermilion is thus an important factor affecting interpersonal impressions and face-to-face communication. This makes it an attractive subject for dermatological investigators, particularly in the field of aesthetics. Notably, the redness of the vermilion decreases significantly with age [4].

The red color of the vermilion is thought to be primarily due to the presence of oxygenated blood, though the literature on this topic is limited. The vascular patterns in the skin of the upper lip (intermediate skin zone between the nose and the upper vermilion border) and lower lip (skin zone just beneath the lower vermilion border) have been studied ultrasonographically [5]. In addition, macroscopic observations of the locations of the arteries throughout the entire lip have also been reported, but those studies focused only on the larger arteries situated deeper in the tissue, not on the superficial blood vessels in the vermilion [6]. Neither of those studies examined the blood vessels in the lip vermilion or the effect of aging on those vessels. To our knowledge, only a single study has addressed the issue of blood flow in the vermilion and the effect of aging [4]. Those investigators used noninvasive measurements of spectral reflectance to determine that the erythema index in the lower vermilion, which reflects the hemoglobin levels there, decreases with aging. There is thus little available histological or anatomical information on the lip vermillion, despite its importance to facial appearance. Moreover, the blood vessels in the vermilion are fundamental to the general function and maintenance of the lip as an organ. For these reasons, the blood vessels of the lip vermilion are worth investigating. The present study provides histological evidence of age-dependent deterioration of the vessels in the upper lip vermilion.

## RESULTS

**Figure 1:**
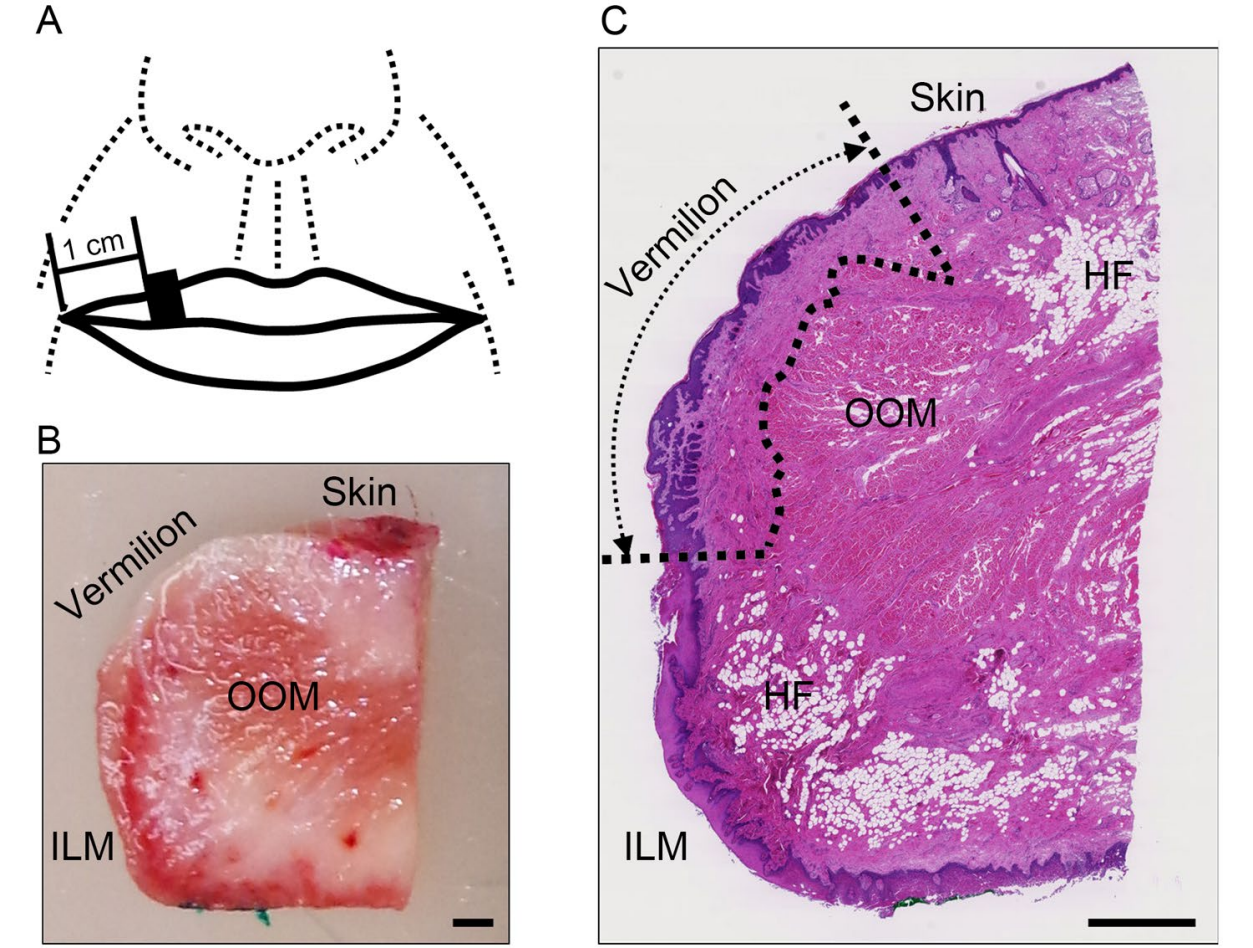
**Areas used for investigation.** (**A**) Specimen collection site: the black square indicates the site of specimen collection. (**B**) Representative image of a dissected specimen: the skin surface and intraoral labial mucosa on the oral cavity side were stained with red and green pigments, respectively. Bar=1 mm; OOM, orbicularis oris muscle; ILM, intraoral labial mucosa. (**C**) Representative image of hematoxylin and eosin stained section of upper lip: the boundary of the vermilion is indicated by the dotted line. Bar=1 mm; OOM, orbicularis oris muscle; HF, hypodermal fat; ILM, intraoral labial mucosa. Note: Hypodermal fat in the vermilion was absent from all specimens.

Upper lip tissues were dissected and sagittally sectioned, after which the following anatomical structures were analyzed. The vermilion zone was detected as the intermediate zone between the lip skin, which contains the keratinized epidermis, hair follicles, sebaceous glands and the hypodermal fat, and the intraoral labial mucosa, which contains the hypodermal fat and the glandulae labiales on the side beyond the rima oris. This definition is consistent with the vermilion zone confirmed by dye staining from the surface before dissection (Figure 1).

### Negative correlation between blood vessels in the vermilion dermis and age

**Figure 2:**
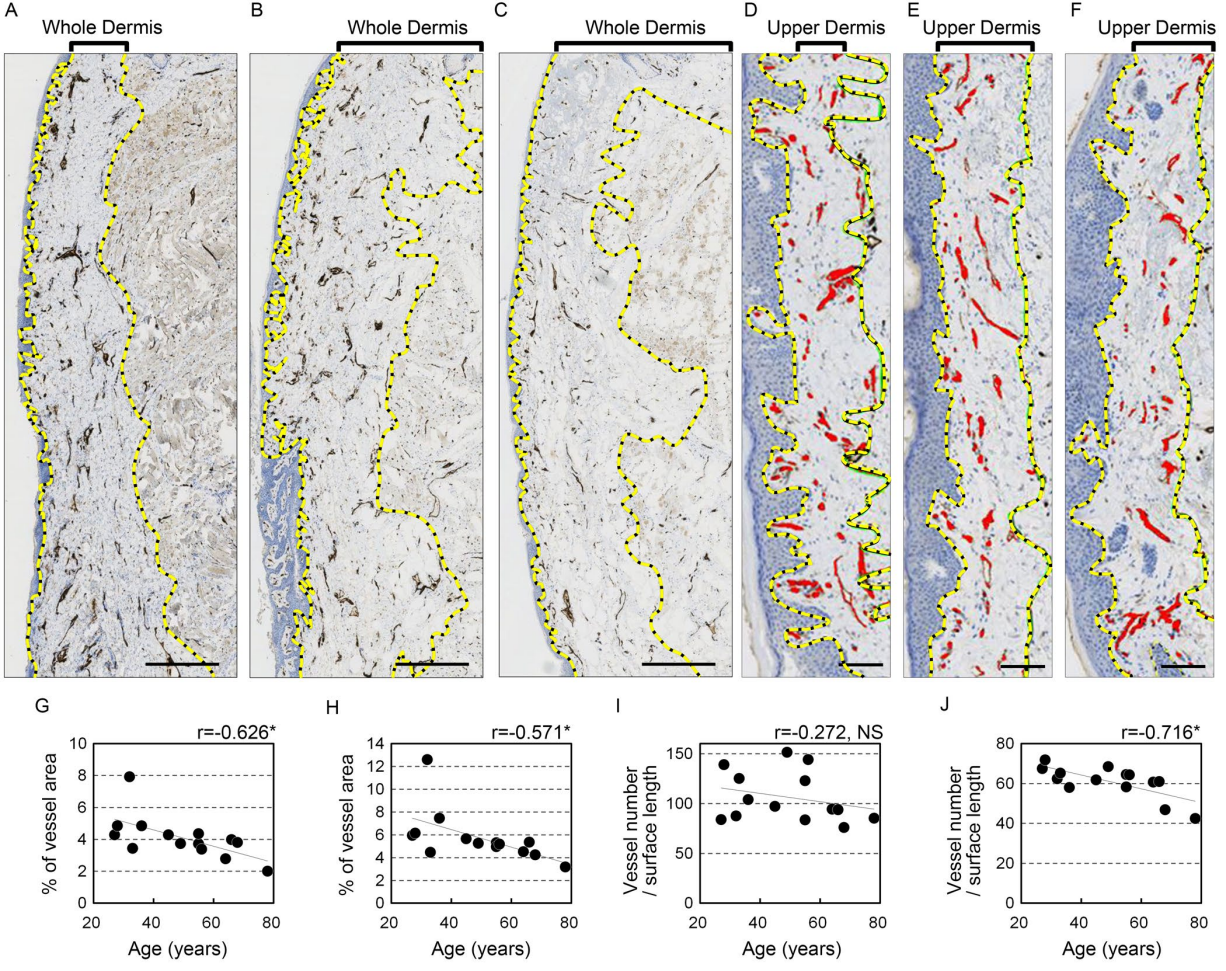
**Blood vessels in the upper lip vermilion dermis decrease with age.** Vessels are stained for anti-CD31. (**A**-**F**) Representative image staining: brown, CD31+ vessels; blue, cell nuclei. (**A**-**C**) Images of the whole vermilion dermis. Donors were 28 (**A**), 49 (**B**) and 64 (**C**) years old. Bar=500 μm. The yellow dotted line marks the boundary line of the whole dermis. (**D**-**F**) Images of the upper dermis of the vermilion. Donors were 27 (**D**), 45 (**E**), and 68 (**F**) years old. Bar=100 μm; red, blood vessels in upper dermis of the vermilion; yellow dotted line, boundary for the upper dermis. (**G**-**K**) Individual plots of vessel parameters shown according to age. (**G**, **H**) Age plotted against percentage of blood vessel area in the analyzed area. (**I**, **J**) Age plotted against the number of blood vessels per surface length (mm). (**G**, **I**) Whole vermilion dermis. (**H**, **J**) Upper dermis of the vermilion. n=14. *p<0.05 (Pearson's correlation test).

To determine whether blood vessels in the vermilion change with age, immunohistochemical staining for CD31 (platelet-endothelial cell adhesion molecule-1) was performed. As expected, the anti-CD31 antibody specifically labeled blood vessels (Figure 2A-F). Moreover, the statistical analysis showed that the area occupied by blood vessels in each section significantly decreased with advancing age in both the whole (r=-0.626, p=0.017) and upper (r=-0.571, p=0.033) dermis (Figure 2G-H, Table 1). This age-dependent decline in blood vessel area was confirmed when calculated relative to surface length in both the whole (r=-0.681, p=0.007) and upper (r=-0.682, p=0.007) dermis (Table 1). By contrast, there was no significant correlation between age and the number of blood vessels in the whole dermis (Figure 2I, Table 1). However, the number of blood vessels per surface length decreased substantially with age in the upper dermis (r=-0.716, p=0.004; Figure 2J, Table 1), though there was no significant change vessel number relative to dermal area in the upper dermis (Table 1). These results indicate that blood vessels in the upper lip vermilion attenuate with age. The mode of this attenuation may be shortening of the blood vessels extending toward the upper dermis without a change in the total number in the whole dermis.

**Table 1:** Correlations between blood vessel parameters and age.

Parameters	Correlation coefficient (r)	p
Vessels in whole dermis		
area/dermal area (%) area/surface length (%)	-0.626	0.017*
area/surface length (%)	-0.681	0.007*
number/surface length (mm)	-0.272	0.347
number/dermal area (mm^2^)	-0.014	0.963
Vessels in upper dermis (~200 µm)		
area/dermal length (%)	-0.571	0.033*
area/surface area (%)	-0.682	0.007*
number/surface length (mm)	-0.716	0.004*
number/dermal area (mm^2^)	-0.349	0.221


n=14, * significant correlation

### Morphological evaluation of stratified squamous epithelium

**Figure 3:**
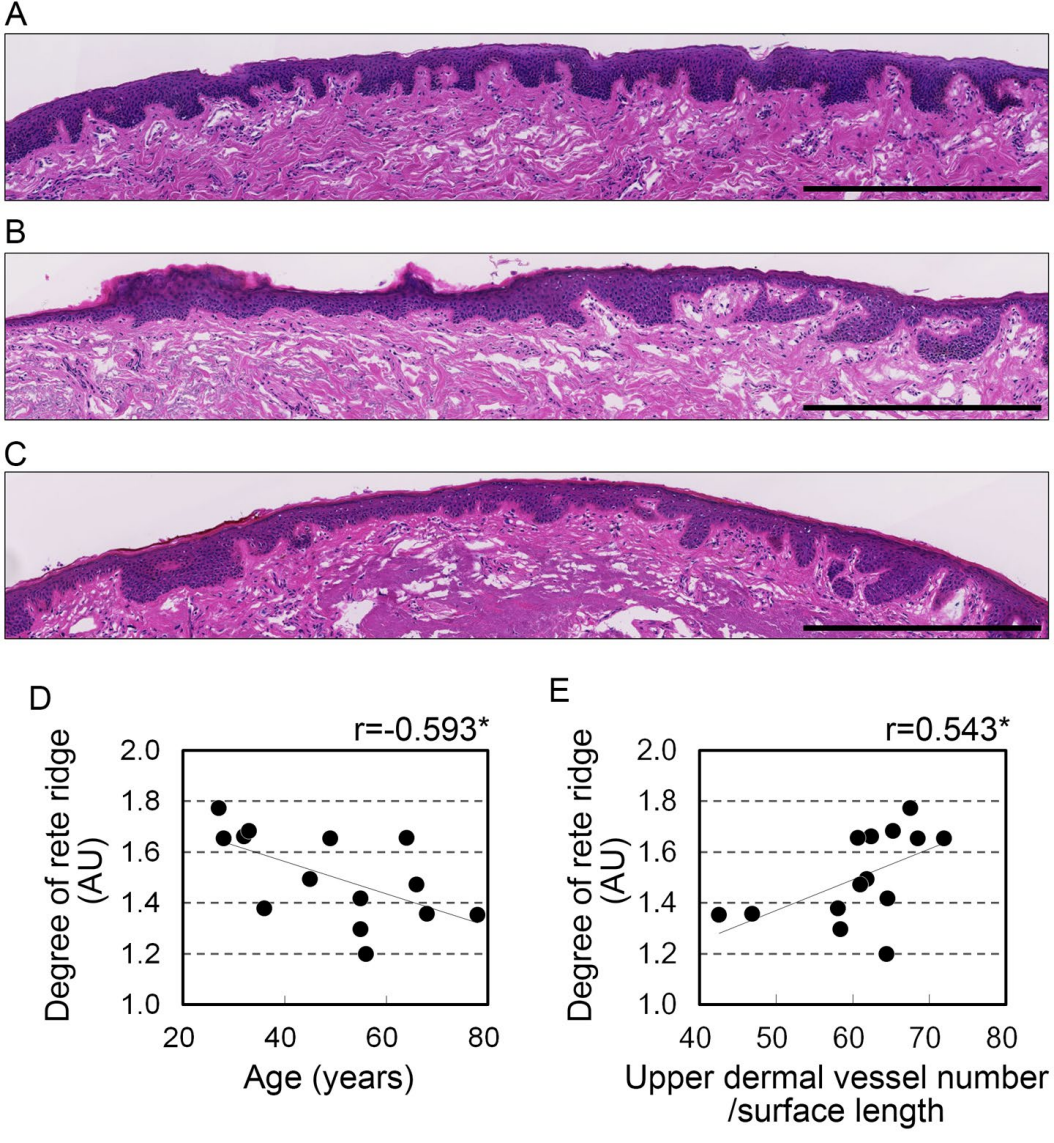
**Development of rete ridges correlates with age and vascular condition in the upper dermis.** (**A**-**C**) Representative images from hematoxylin and eosin staining in the upper dermis of 28 (**A**), 45 (**B**), and 68 (**C**) year old donors. Bar=500 μm. (**D**, **E**) Rete ridge elongation plotted against (**D**) age and (**E**) number of blood vessels in the upper dermis per surface length. n=14. *p<0.05; NS, not significant (Pearson's correlation test).

The histological structure of the stratified squamous epithelium can affect the color of lips seen from the surface. This is because light is reflected by the blood vessels underlying this layer. To investigate the influence of stratified squamous epithelium on lip color, its morphology was histologically examined. The findings are summarized in Table 2. The surface length of the vermilion in sagittal sections tended to be shortened with advancing age, but this change was not significant (r=-0.524, p=0.055). The mean the thickness of the stratified squamous epithelium and the length of the dermo-epithelial junction did not correlate with age. By contrast, epithelial extensions into the dermis, calculated as rete ridge elongation, correlated negatively with age (r=-0.593, p=0.026, Figure 3A-D), which indicates an age-related flattening of the dermo-epithelial junction. This flattening without a change in epithelial thickness suggests there is a diminishing of the wavy structure and variation in thickness of the stratified squamous epithelium, resulting in a more uniform intermediate thickness.

**Table 2:** Correlations between morphological parameters in stratified squamous epithelium and age.

Parameters		Correlation coefficient (r)	p
Correlation to donor age			
a)	surface length (µm)	-0.524	0.055
b)	mean thickness (µm)	-0.403	0.152
c)	length of dermo-epithelial junction (µm)	-0.404	0.153
d)	rete ridge elongation (c/a) (AU)	-0.593	0.026*


n=14, * significant correlation

### Number of blood vessels in the upper dermis correlates with rete ridge elongation

Dermal papillae, which are located on the opposite side of the dermis from the rete ridges, generally contain blood capillaries. This suggests blood vessels may contribute to the state of the rete ridges. Analysis of the correlation between the blood vessels and rete ridges revealed a significant positive correlation between the number of blood vessels in the upper dermis per surface length and rete ridge elongation (r=0.543, p=0.044; Figure 3E, Table 3). However, the number of blood vessels in the whole dermis and the area occupied by blood vessels did not correlate with rete ridge elongation (Table 3).

**Table 3:** Correlation between rete ridge elongation and blood vessel parameters.

Parameters		Correlation coefficient (r)		p		
Correlation between rete ridge elongation and vessel parameters					
Vessels in whole dermis					
	area/surface length (%)	0.261			0.368
	area/dermal area (%)	0.306			0.288
	number/surface length (mm)	-0.011			0.971
	number/dermal area (mm^2^)	-0.010			0.974
Vessels in upper dermis (~200 µm)					
	area/surface length (%)	0.331	0.248		
	area/dermal area (%)	0.285	0.323		
	number/surface length (mm)	0.543	0.044*		
	number/dermal area (mm^2^)	0.360	0.206		


n=14, * significant correlation

### Cell proliferation in stratified squamous epithelium

**Figure 4:**
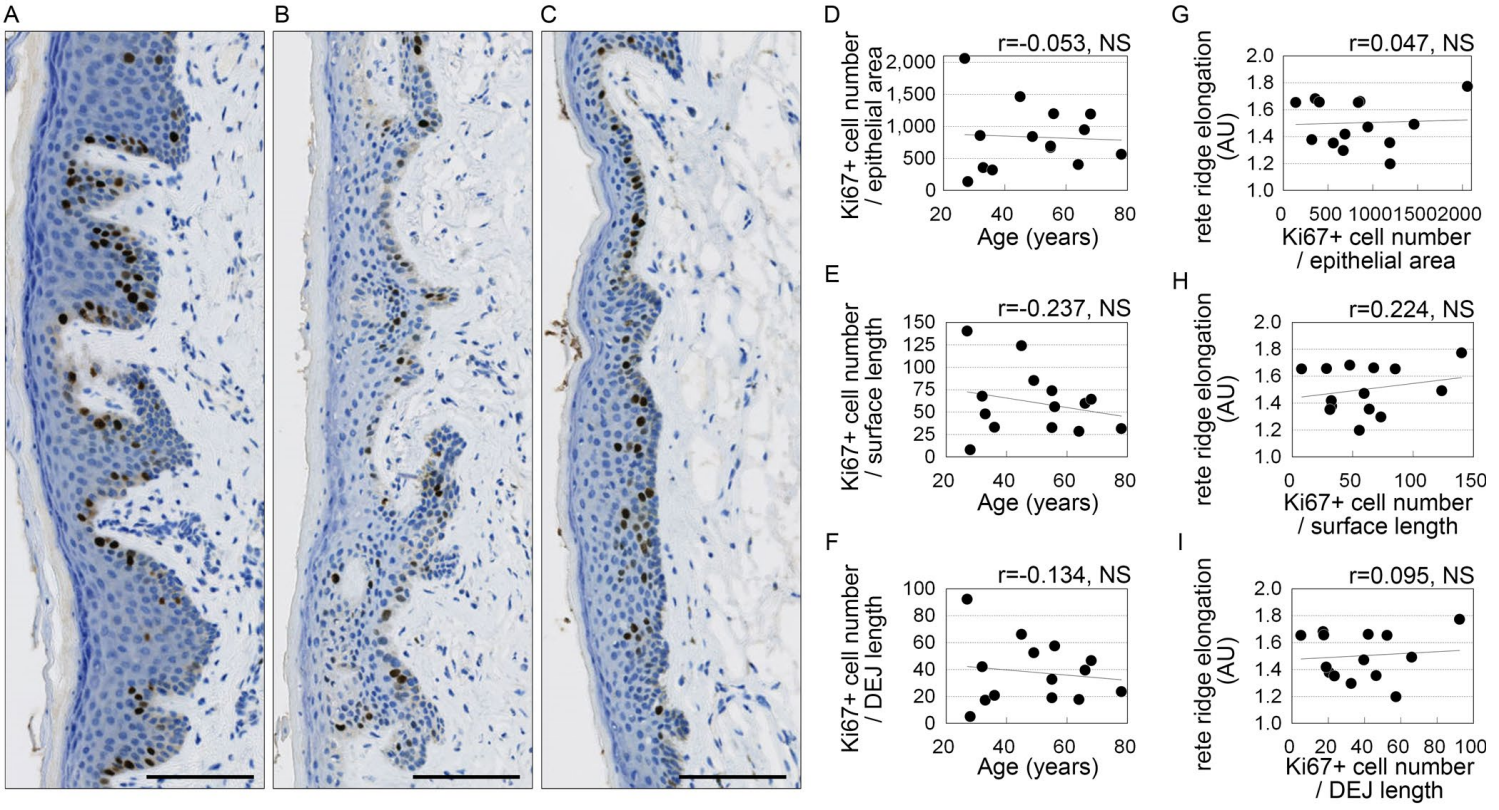
**Squamous epithelial cell proliferation of does not correlate with age or rete ridge elongation.** (**A**-**C**) Representative images of Ki67 staining. Donors were 32 (**A**), 49 (**B**), and 78 (**C**) years old. Bar=100 μm; brown, Ki67+ cells; blue, cell nuclei. (**D**-**I**) Numbers of Ki67-positive cells plotted against age (**D**-**F**) or rete ridge elongation (**G**-**I**). (**D**, **G**) Numbers of Ki67-positive cells per area of stratified squamous epithelium area (number/mm^2^). (**E**, **H**) Numbers of Ki67+ cells per surface length (number/mm). (**F**, **I**) Numbers of Ki67+ cells per length of the dermo-epithelial junction (DEJ) (number/mm). n=14; NS, not significant (Pearson's correlation test).

Epithelial extensions produced through proliferation of stratified squamous epithelial cells are thought to contribute to the formation of rete ridges [7–10]. Therefore, to investigate the influence of proliferative activity on the flattening of the dermo-epithelial junction, cells in the stratified squamous epithelium were immunostained for anti-Ki67, a marker of cell proliferation. The number of Ki67-positive cells did not correlate significantly with age or rete ridge elongation relative to the area or surface length of the stratified squamous epithelium, or to the length of the dermo-epithelial junction (Figure 4).

## DISCUSSION

The present study demonstrated overall deterioration of the blood vessels in the upper lip dermis with aging. The results are in good agreement with the overall change in cutaneous blood vessels with age [11], and show the influence of vascular alterations similar to those in skin [12]. More specifically, both the area and number of blood vessel in the dermis of the upper lip vermilion decreased with age relative to vermilion surface length. Blood vessel area was also decreased relative to dermal area, but the number of blood vessels per dermal area did not correlate with age. Nonetheless, the number of blood vessels decreased markedly in the upper dermis. In facial skin, chronic photodamage leads to gradual, age-related decreases the number and area of dermal vessels as compared buttock skin, which suffers no photodamage [13]. As the lips protrude from around the center of the lower face and are frequently exposed to sun, similar sun-induced alterations likely occur in the upper lip vermilion. Thus, the age-dependent decreases in blood vessels detected in the present study may reflect photoaging processes.

In the present study, the number of vessels per surface length in the dermis of the upper vermilion showed prominent decreases with age, but did not correlate with age in the whole dermis. This difference reflects the nature of the skin vasculature. In studies comparing the number of capillary loops visible in the skin using videocapillaroscopy versus the blood flux in the skin determined using laser Doppler flowmetry [14], the number of capillary loops (reflecting blood vessels in the upper dermis of the skin) decreased significantly with age, whereas blood flux in the skin, which included vessels in the middle and deeper layers of the dermis, was significantly increased [14]. Changes in the upper lip vasculature are thus similar to those in skin, and our present results for the upper lip vessels should be helpful for explaining discrepancies in the literature [14]. Furthermore, this reduction in the vasculature implies a decrease in superficial dermal blood, which would influence lip color and could help explain the earlier reported age-related decrease in the hemoglobin index [4]. Considering that vascularity affects the redness of the skin surface in general, the mechanism by which redness in the lips decreases with age can be inferred from the results of the present study. However, in contrast to the lips, redness of the skin reportedly increases with age [14]. The causes of this difference remain unclear, but one possible explanation is that blood flow in the deeper part of the dermis has less impact on the color of the lips than the skin. Another possibility is that increased blood flow in the deeper dermis does not occur in the lip dermis. Further investigation will be necessary to clarify the reasons for this discrepancy.

The present study revealed a flattening of the rete ridges with age. This flattening correlated with the number of blood vessels in the upper dermis, but not with the number of Ki67-positive cells in the stratified squamous epithelium. Previous reports suggest epithelial cell proliferation is involved in the elongation of the rete ridges in the skin [7–10]. On the other hand, in the oral mucosa, physical force appears to cause the formation of rete ridges [15]. Blood vessels may also contribute to the formation of rete ridges, as the dermal papillae on the dermal side of the rete ridges contain capillaries [16,17], which may correlate with ridge length [18]. Our correlational findings suggest blood vessels rather than epithelial cell proliferation contributes to the formation of rete ridges, though they can provide little insight into the mechanism.

Several limitations must be considered in the present study. First, because the specimens were isolated from the deceased and then frozen, deformation of tissues during freezing/dissection as well as possible degradation of antigens before excision of the tissue from the donor cannot be excluded. To overcome these potential problems, we expressed measurements per unit area or per unit length. Second, the background characteristics of the lip donors, such as their medical history, whether they smoked, their sun exposure frequency, and dietary habits were uncontrolled. Because these background characteristics can potentially influence the condition of the blood vessels [12], confounding factors may exist between the vascular condition and donor background. Third, there were 14 cases in this study, which was insufficient to reach a generalizable conclusion. Further investigation of additional cases will be necessary to overcome this problem. A final limitation is the analytical methods applied. Immunohistochemical analysis of tissue sections, as conducted in this study, is very useful and reliable but provides a limited view of the vascular morphology. Blood vessels form three-dimensional (3D) branching networks within the dermis, and the present study may therefore have overlooked some alterations. Reports using emerging techniques for improving optical clarity of tissue specimens, including BBAB, CUBIC and iDISCO, have been increasing [19–21]. These methods would enable us to clarify the 3D morphology of blood vessels from both the internal side of the specimen and from its surface, with a spatial resolution on the order of millimeters. However, many specimens would be needed, and they are difficult to obtain through biopsy from healthy individuals. New optical imaging techniques, including optical coherence tomography angiography, reflectance confocal microscopy, and two-photon autofluorescence lifetime imaging, offer new, noninvasive approaches to making 3D observations of blood vessels [16,17,22], though the measurement depth is shallow. The widespread adoption of such new approaches will help us to better understand the influence of blood vessels in the lip on its appearance.

In summary, to the best of our knowledge, this study provides the first histological evidence of age-related decreases in blood vessels in the upper lip vermilion. Our findings provide new information on the lip vermilion and new insight into how the dermal vasculature affects the vermilion as individuals age. These results could serve as the basis for development of new lip treatments for older individuals.

## MATERIALS AND METHODS

### Specimens

Human upper lip specimens from 14 Caucasian female cadavers (age range 27-78 years old) were kindly provided by Obio, LLC (El Segundo, CA) while complying with ethics and all applicable laws, rules and regulations. It was confirmed prior to the study that written informed consent had been obtained from each donor by Obio, LLC. All specimens were identified with a randomly assigned 6-digit alphanumeric code during experiments. The use of human tissue specimens was approved by the institutional review board of POLA Chemical Industries on 22 June 2017.

### Antibodies

Monoclonal mouse anti-human CD31 antibody (clone JC70A), monoclonal mouse anti-human CD34 antibody (clone QBEnd/10), monoclonal mouse anti-human Ki67 antibody (clone MM1), negative mouse isotype control antibody, and Bond Polymer Refine Detection were purchased from Leica Biosystems (Newcastle, UK). All antibodies were provided in ready-to-use condition.

### Preparation of sample

The vermilion of the upper lip was dissected from 1 cm inside from the right corner of the mouth to a width of 3 mm, then fixed in 10% neutral-buffered formalin and embedded in paraffin. Sagittal 4-μm-thick microtome sections (in the direction from the vermilion surface toward the nose) were immunohistochemically stained for CD31, CD34 and Ki67, and also stained with hematoxylin and eosin.

### Immunohistochemistry

After deparaffinization and rehydration, sections were stained with each antibody using the procedure recommended by the manufacturer. Briefly, the sections were incubated first with hydrogen peroxide to quench endogenous peroxidase activity and then with diluted normal rabbit serum to reduce nonspecific reactions. Thereafter, the sections were incubated with each of the primary antibodies listed above and then with Post Primary IgG linker reagent (Leica Biosystems) and Poly-HRP IgG reagent (Leica Biosystems). The labeling was visualized as brown staining using 3,3'-diaminobenzidine tetrahydrochloride (DAB). Hematoxylin counterstaining was also performed to visualize cell nuclei.

### Image analyses

Stained sections were digitized using a NanoZoomer-XR slide scanner system (Hamamatsu Photonics, Hamamatsu, Japan) and analyzed using WinROOF image-processing software (Mitani Corporation, Fukui, Japan). CD31-immunostained images were used for analyzing blood vessels because similar images were obtained with both CD31 and CD34 immunostaining. A region of CD31-positive staining with an area >25 mm^2^ was considered to be a blood vessel. The number of vessels and percentage of the whole dermis and upper dermis (to a depth of 200 μm from a dermo-epithelial junction) occupied by vessels were calculated as parameters. The whole dermis was defined as the area between the dermo-epithelial junction and the external surface of the orbicularis oris muscle in the vermilion zone. Morphological characteristics of the stratified squamous epithelium were measured as follows: surface length as the external contour of the living cell layer of the stratified squamous epithelium; length of the dermo-epithelial junction as the internal contour of stratified squamous epithelium; mean thickness as the area of the living cell layer of the stratified squamous epithelium divided by the surface length; and rete ridge elongation as the ratio of the length of the dermo-epithelial junction to the surface length. The number of Ki67-positive cells in the stratified squamous epithelium was counted using the cell-counting function (Ki67) in WinROOF software.

### Statistics

The R package was used for all statistical analyses (the R Project, http://www.R-project.org). Pearson's correlation coefficient was used for correlation analyses, and values of P < 0.05 were considered significant.
